# Modeling cell-mediated immunity in human type 1 diabetes by engineering autoreactive CD8^+^ T cells

**DOI:** 10.3389/fimmu.2023.1142648

**Published:** 2023-05-30

**Authors:** Leeana D. Peters, Wen-I Yeh, Juan M. Arnoletti, Matthew E. Brown, Amanda L. Posgai, Clayton E. Mathews, Todd M. Brusko

**Affiliations:** ^1^ Department of Pathology, Immunology and Laboratory Medicine, College of Medicine, Diabetes Institute, University of Florida, Gainesville, FL, United States; ^2^ Department of Pediatrics, College of Medicine, Diabetes Institute, University of Florida, Gainesville, FL, United States

**Keywords:** CD8 T cell, T cell receptor knockout, type 1 diabetes, gene editing, autoimmunity

## Abstract

The autoimmune pathogenesis of type 1 diabetes (T1D) involves cellular infiltration from innate and adaptive immune subsets into the islets of Langerhans within the pancreas; however, the direct cytotoxic killing of insulin-producing β-cells is thought to be mediated primarily by antigen-specific CD8^+^ T cells. Despite this direct pathogenic role, key aspects of their receptor specificity and function remain uncharacterized, in part, due to their low precursor frequency in peripheral blood. The concept of engineering human T cell specificity, using T cell receptor (TCR) and chimeric antigen receptor (CAR)-based approaches, has been demonstrated to improve adoptive cell therapies for cancer, but has yet to be extensively employed for modeling and treating autoimmunity. To address this limitation, we sought to combine targeted genome editing of the endogenous TCRα chain gene (*TRAC*) via CRISPR/Cas9 in combination with lentiviral vector (LV)-mediated TCR gene transfer into primary human CD8^+^ T cells. We observed that knockout (KO) of endogenous *TRAC* enhanced *de novo* TCR pairing, which permitted increased peptide:MHC-dextramer staining. Moreover, *TRAC* KO and TCR gene transfer increased markers of activation and effector function following activation, including granzyme B and interferon-γ production. Importantly, we observed increased cytotoxicity toward an HLA-A*0201^+^ human β-cell line by HLA-A*02:01 restricted CD8^+^ T cells engineered to recognize islet-specific glucose-6-phosphatase catalytic subunit (IGRP). These data support the notion of altering the specificity of primary human T cells for mechanistic analyses of autoreactive antigen-specific CD8^+^ T cells and are expected to facilitate downstream cellular therapeutics to achieve tolerance induction through the generation of antigen-specific regulatory T cells.

## 1 Introduction

Autoreactive T cells specific for β-cell antigens have been shown to be integral to type 1 diabetes (T1D) disease pathogenesis in the non-obese diabetic (NOD) mouse and are associated with human disease progression through immune studies and histopathological evidence (1). Murine models of T1D have helped to illuminate numerous antigens targeted during the initiation and propagation of the autoimmune process (2–4). One of these antigens, islet-specific glucose-6-phosphatase catalytic subunit-related protein (IGRP), has been implicated as a key autoantigen in the non-obese diabetic (NOD) mouse model of T1D (5). Moreover, landmark human tissue studies have visually confirmed the targeting of this and other antigens by CD4^+^ and CD8^+^ T cells *in situ* in T1D with peptide-MHC multimer staining (6).

In order to understand the contribution of autoreactive T cells in driving T1D, we previously generated islet antigen-specific T cell “avatars” via lentiviral-mediated TCR gene transfer to primary CD8^+^ T cells. Our previous work using the IGRP_265-273_ reactive (clone 32) (7) T cell “avatars” highlighted the role of innate cytokines IL-12 and IL-18 to synergistically drive a Tc1 phenotype (8), which enhanced the killing of the β-cell line βlox5 (9). Moreover, our group has also utilized this clone to investigate the regulatory mechanism underlying type I interferon-mediated T cell cytotoxicity (10). Importantly, we demonstrated the utility of these avatars in constructing an isogenic system to investigate key disease relevant cellular interactions (11). Despite these advances in assessing antigen-specific T cell function, there remain a number of outstanding questions regarding which receptor specificities are directly pathogenic and thus, important for T1D initiation and progression. Our lab (12) and others (13, 14) have previously demonstrated that despite some repertoire sharing across tissues (up to 24% of clones), the CD8^+^ T cell repertoire in islet infiltrates and secondary lymphatics remains remarkably diverse, with reactivities against both native and post-translationally modified antigens detected (14). Importantly, the vast majority of CD8^+^ T cell lines from handpicked islets of a recent onset T1D donor were of unknown reactivities (14), highlighting the need for studies examining a broader range of autoantigen targets and immune receptors in T1D. There is also increasing evidence of divergent patterns of initial targets of autoimmunity, characterized by insulin autoantibody (IAA) or GAD autoantibody (GADA) initial seroconversion, which seems to be linked to age (15) as well as genetic risk at the HLA region (16) (HLA-DR4 and HLA-DR3, respectively). Thus, these potential disease endotypes, coupled with the large impacts of environmental exposures and aging on the immune repertoire (17, 18), as well as heterogeneity in immune phenotypes of CD8^+^ T cells in T1D (19), all serve to complicate the assessment of antigen-specific phenotype and function.

*In vitro* modeling of autoimmunity and clinical adoptive cell therapy (ACT) strategies rely on the generation of large numbers of autoreactive T cells, surpassing the frequency obtainable in peripheral blood without extended months of clonal expansion, potentially altering the phenotype from the *in vivo* state. Despite the success of common methods to generate antigen-specific T cells (20), namely using a viral (often lentiviral) vector to deliver a *de novo* T cell receptor (TCR), there remain concerns of off-target effects arising from heterologous TCR chain pairing (21, 22). Work in the cancer immunotherapy space have demonstrated the utility of gene editing technologies (i.e., CRISPR/Cas9) in targeting endogenous TCRs to enhance on target-specificity and anti-tumor functionality (21, 22), though to our knowledge this has not been shown previously for T1D autoantigen reactive T cells. We hypothesized that reducing the potential for heterologous chain pairing would translate into enhanced effector phenotype and function of a T1D-associated IGRP_265-273_ reactive clone (clone 32) (7). We chose to test our workflow in engineered IGRP-reactive CD8^+^ T cells, as a number of studies have explored the importance of this antigen in T1D as a target for human CD8^+^ T cells in circulation (5, 23) and *in situ* (6). Therefore, we used CRISPR/Cas9 to knockout (KO) the endogenous TCRα in engineered IGRP-reactive CD8^+^ T cells, with the goal of more effectively recapitulating autoreactive CD8^+^ T cell function in T1D.

## 2 Materials and methods

### 2.1 Human subjects

Peripheral blood mononuclear cells (PBMCs) were isolated via density-gradient centrifugation from leukapheresis-processed blood of healthy donors (N=4, median age: 25.5 years, range 21-27 years, 25% male) purchased from LifeSouth Community Blood Centers (Gainesville, FL).

### 2.2 Cell isolation and expansion

Naïve CD8^+^ T cells were isolated in a two-step procedure consisting of negative selection followed by positive selection from leukapheresis products using the naïve CD8^+^ T cell isolation kit (Miltenyi Biotec) and were cultured as reported previously (9). Briefly, cells were seeded at a concentration of 2.5 x 10^5^ cells/mL in complete RPMI formulated as previously described (9) and stimulated with α-CD3/28 coated beads (Dynabeads, Thermo Fisher) at a 1:1 ratio with 100 IU/mL recombinant human IL-2 (Teceleukin, NIH). Media and IL-2 were added assuming consumption every 2-3 days, with 5 ng/mL IL-7 (R&D systems) added every 2-3 days after day 5 of culture.

### 2.3 Generation of autoreactive T cell avatars

Cells were transduced 48 hours post-activation with a multicistronic (pCCL.TRB.P2A.TRA.T2A.eGFP) lentiviral vector (LV) encoding the HLA-A*02:01 restricted IGRP reactive clone 32 TCR (7) or mock (pCNFW.eGFP) control LV as previously described (24, 25). Briefly, virus was added at 3 transducing units (TU)/cell along with 8 µg/mL protamine sulfate, and cells were spinnoculated at 1000x*g* for 30 minutes at 32°C. Transduction efficiency was 34.0±4.1%. Cells were expanded, using methods described above, for 5-7 days post-transduction, then assessed by flow cytometry for stable transduction by expression of GFP. Data were acquired on an LSRFortessa flow cytometer (BD Biosciences) and analyzed using FlowJo software v10 (BD).

### 2.4 CRISPR/Cas9-mediated KO of *TRAC*

After 5-7 days of expansion post-transduction, α-CD3/28 coated beads were removed, and transduced T cells were electroporated with ribonucleoprotein (RNP) complexes comprised of a single guide RNA (sgRNA, Synthego) and Cas9 (Thermo Fisher Scientific) at a 6:1 molar ratio (26) (assembled at room temperature for 10 minutes) using the Lonza 4D-Nucleofector X Unit and P3 Primary Cell 4D-Nucleofector X Kit (Lonza Bioscience) with the pulse code EH100. For the negative control condition, cells were electroporated without sgRNA or Cas9. The sequence for the *TRAC* guide used was as follows: GUCAGGGUUCUGGAUAUCUG.

### 2.5 Confirmation of TCRα KO and MHC-multimer binding

Efficiency of *TRAC* KO was assessed 5-7 days post CRISPR/Cas9 (culture day 12-14). Briefly, cells were stained with Live/Dead Fixable Near-IR (Thermo Fisher Scientific) for 10 minutes at 4°C, washed with stain buffer (PBS + 2% FBS + 0.05% NaN_3_), and subsequently stained for 30 minutes at 4°C with anti-human CD3e-PerCP-Cy5.5 (clone UCHT1, BioLegend) and TCRαβ-BV421 (clone IP26, BioLegend) for 30 minutes at 4°C. Endogenous TCRα KO efficiency was assessed by percentage of TCRαβ negative cells within the internal control untransduced (GFP-) cells for each donor culture. To assess changes in alpha chain expression and proper *de-novo* TCR chain pairing, cells were stained with Live/Dead Fixable Near-IR as above in addition to CD8-BV605 (clone SK1, BioLegend), TCR V alpha 2 (V⍺2, clone F1, Thermo Fisher) conjugated in house using the Zenon Alexa Fluor 647 mouse IgG2a labeling kit (Invitrogen) according to manufacturer instructions, and TCR V beta 20-1-PE (Vβ2, clone MPB2D5, Beckman Coulter) or TCRαβ-BV421 (clone IP26, BioLegend) for 30 minutes at 4°C. To assess changes in functional receptor avidity, IGRP_265-273_ MHC class I dextramer staining was conducted as per manufacturer’s instructions (Immudex). Briefly, cells were stained with fixable Live Dead NIR as above, followed by incubation with TruStain Fc block (BioLegend) for 5 minutes and incubation with 10 µL dextramer for 10 minutes at room temperature, after which cells were extracellularly stained with anti-human CD8-BV605 (clone SK1, BioLegend) and TCRαβ-BV421 (clone IP26, BioLegend) for 20 minutes at room temperature. Data were acquired on an LSRFortessa flow cytometer (BD Biosciences) and analyzed using FlowJo software v10 (BD).

### 2.6 Stimulation assays

A derivative of the chronic myelogenous leukemia (CML) cell line K562 engineered to express HLA-A*02:01 (9, 24), kindly provided by Drs. James Riley and Bruce Levine (University of Pennsylvania), was used as a source of artificial antigen presenting cells (aAPCs) for our T cell avatars. K562s were loaded with 10 µg/mL IGRP_265-273_ peptide (GenScript) at a concentration of 1 x 10^6^cells/mL at 4°C for one hour. IGRP TCR transduced (IGRP) or eGFP mock transduced T cells (mock), with or without *TRAC* KO, were plated at a 1:1 ratio with peptide-pulsed K562s. Cells were stained to assess expression of activation markers using Live/Dead Fixable Near-IR as above, after which cells were incubated with TruStain Fc block (BioLegend) for 5 minutes at 4°C. Cells were stained for 30 minutes at 4°C with anti-human CD69-BV711 (clone FN50, BioLegend) and CD25-APC (clone BC96, BD Biosciences) at baseline and after 2, 4 and 24 hours of stimulation. To assess the cytokine production profile, cells were stimulated as above for 4 hours at 37°C in the presence of 0.66 µL/mL Golgistop (BD Biosciences), after which cells were stained with anti-human CD8a-APC (clone RPA-T8, BD Biosciences) and anti-GFP-AF488 (clone FM264G, BioLegend) prior to fixation and permeabilization with FOXP3 transcription factor staining buffer set (eBioscience) according to manufacturer’s protocol. Intracellular staining was performed for 30 minutes at room temperature with anti-human GZMB-PE (clone GB11, BD Biosciences), TNFα-BV650 (clone MAb11, BioLegend), Perforin-BV421 (clone dG9, BioLegend), IFNγ-PE-Cy7 (clone 4S.B3, BioLegend), and IL-2-PerCP-Cy5.5 (clone MQ1-17H12, BioLegend).

### 2.7 Cell-mediated lympholysis

*TRAC* KO or electroporation only negative control IGRP_265-273_ reactive CD8^+^ T cells were assayed for their capacity to kill the HLA-A*02:01-expressing human β-cell line βLox5, as previously described (9). In brief, βLox5 cells were harvested from flasks by incubating with Cell Dissociation Buffer for 5-10 minutes at 37°C and 5% CO_2_ (Gibco). βLox5 cells were then washed, resuspended at 5-10 x 10^6^ /mL in PBS, and labeled with 5uM CellTrace Violet (Thermo Fisher Scientific) according to manufacturer’s instructions. Labeled βLox5 were plated at a concentration of 30,000 cells per well in a 48-well tissue culture treated plate in complete DMEM (cDMEM) formulated as previously described (27). After 24hr, CD8^+^ T cell avatars were plated with βLox5 at 0:1, 1:1, and 5:1 effector:target (E:T) ratios in duplicate, and co-cultured for 16 hours at 37°C and 5% CO_2_. Cells were harvested and stained with a master mix comprised of 88uL Annexin V-Binding Buffer (BioLegend), 2uL Annexin V-AF647 (BioLegend) and 10uL of propidium iodide at 3mg/mL (PI, Invitrogen). Data were acquired on an LSRFortessa flow cytometer (BD Biosciences) and analyzed using FlowJo software v10 (BD).

### 2.8 Statistical analyses

Statistical testing and visualization were performed in GraphPad Prism Software V9. Data were analyzed by Two-Way ANOVA with Bonferroni correction or Tukey’s multiple comparison test and paired t test as described in the figure legends and text. Data are represented as mean ± standard deviation (SD) unless otherwise specified. P-values <0.05 were considered significant.

## 3 Results

### 3.1 KO of the endogenous TCRα enhances pairing of an engineered T1D-associated TCR

**Figure 1:**
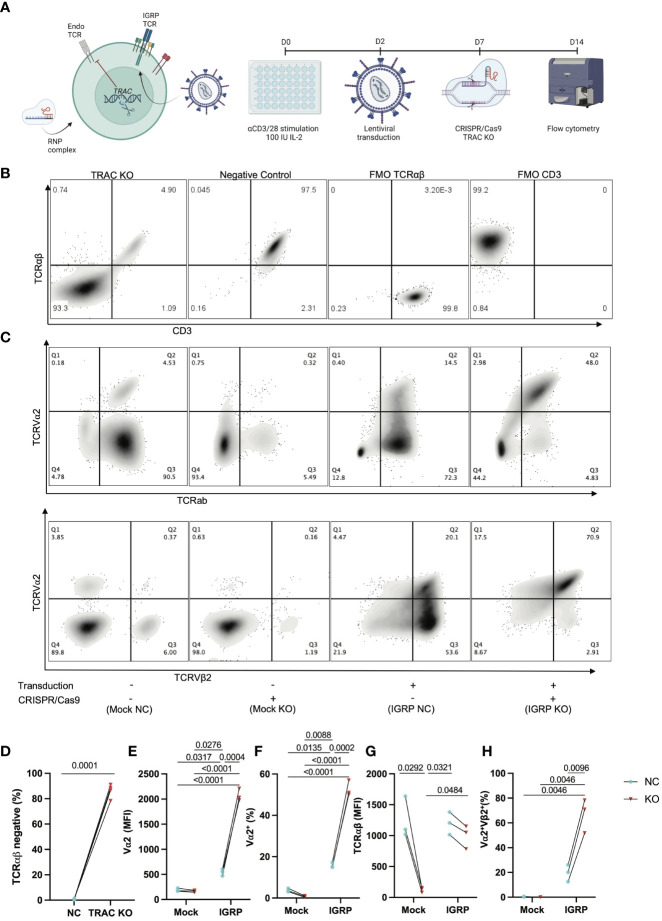
Efficient KO of the endogenous TCR is achieved by targeting *TRAC*. **(A)** Schematic of our experimental procedure wherein CD8^+^ T cells are transduced with a LV encoding the IGRP-reactive clone 32 TCR as reported previously (24), after which CRISPR/Cas9 was performed targeting *TRAC* using RNP complexes comprised of a 6:1 molar ratio of sgRNA : Cas9. Created with BioRender.com. **(B)** Representative plots of TCRαβ and CD3 expression in GFP- cells (internal controls within each donor culture which are exposed to editing conditions but are not stably transduced with the *de novo* TCR) show high efficiency KO as delineated by largely all TCRαβ negative cells (far left) as compared to the electroporation only negative control condition (middle left), as compared to fluorescence minus one (FMO) controls for TCRab (middle right) and CD3 (far right). **(C)** Representative plot of TCRVα2 and TCRαβ expression (top) and paired TCRVα2 and TCRVβ2 expression (bottom) across all conditions (+ or – transduction with the IGRP reactive clone 32 TCR and + or - CRISPR/Cas9 deletion of endogenous *TRAC*). **(D)** The *TRAC* KO condition possesses significantly more cells that are TCRαβ negative (paired t test, n=4, p value shown on figure). **(E)** *TRAC* KO avatars show significantly increased expression of the clone 32 α-chain TCRVα2 (24), as well as **(F)** increased TCRVα2^+^ cells (Repeated Measures two-way ANOVA with Tukey’s multiple comparison test, n=3, p values shown on figures), without a significant increase in **(G)** total TCR expression between clone 32 TCR transduced negative control and KO avatars (Repeated measures two-way ANOVA with Tukey’s multiple comparison test, n=3, p value shown on figure). We also observe an increase in **(H)** correct *de novo* TCR pairing with *TRAC* KO (Repeated measures two-way ANOVA with Tukey’s multiple comparison test, n=3, p value shown on figure).

We hypothesized that deletion of the endogenous TCRα would reduce heterologous pairing with an exogenously introduced T1D-associated IGRP-reactive TCR, thereby improving on-target specificity. Thus, we generated CD8^+^ T cell avatars reactive to IGRP_265-273_ (7) and targeted the TCRα chain constant (*TRAC*) region via CRISPR/Cas9 (**Figure 1A**), as has been previously reported to improve on-target efficacy of avatars specific for a melanoma antigen (22). Our IGRP-TCR construct possesses 4 base pair (bp) mismatches as well as a mutated protospacer adjacent motif (PAM) (26) at the location targeted by sgRNA1 (**Figure S1**), which importantly, prevents cleavage of the *de novo* TCR by Cas9; hence, we selected this guide for our studies. We first validated the efficiency of endogenous *TRAC* KO in primary human CD8^+^ T cells transduced with the IGRP-reactive clone 32 TCR (9, 10). We achieved, on average, an 86% KO efficiency (86.13 ± 5.5) after targeting *TRAC* via CRISPR/Cas9, as quantified by the percentage of TCRαβ negative cells (**Figures 1B, D**). Moreover, we noted increased expression of the clone 32 TCR alpha variable region, TCRVα2 in the *TRAC* KO avatars (IGRP KO (*red triangles*), 2074.0 ± 122.3) relative to those that received electroporation but not sgRNA or Cas9 (IGRP negative control (NC, *blue circles*), 541.7 ± 68.1, fold difference=3.8), and mock transduced cells lacking the *de novo* TCR which received electroporation only (Mock NC (*blue*), 189.0 ± 32.1, fold difference=10.9) or mock transduced cells deleted for *TRAC* (Mock KO (*red*), 163.3 ± 21.7, fold difference=12.7) (**Figures 1C, E**). We also observed a corresponding increase in cells positive for TCRVα2 in the *TRAC* KO avatars (52.7 ± 3.8) relative to the negative control cells (15.8 ± 1.3, fold difference=3.3), and mock transduced negative control (3.8 ± 0.8, fold difference=13.9) and KO (0.7 ± 0.3, fold difference=71.2) cells (**Figures 1C, F**). This increase in TCRα expression occurred independently of differences in total TCRαβ expression between *TRAC* KO and negative control avatars (**Figure 1G**), indicative of increased proper TCR pairing following editing. Indeed, we observed increased proper pairing in the *TRAC* KO avatars (67.0 ± 13.7) relative to negative control avatars (19.6 ± 6.8, fold difference=3.4), while mock controls lacked expression of both chains, when stained with the *de novo* TCR variable chains TCRVα2 and TCRVβ2 (**Figures 1C, H**).

**Figure 2:**
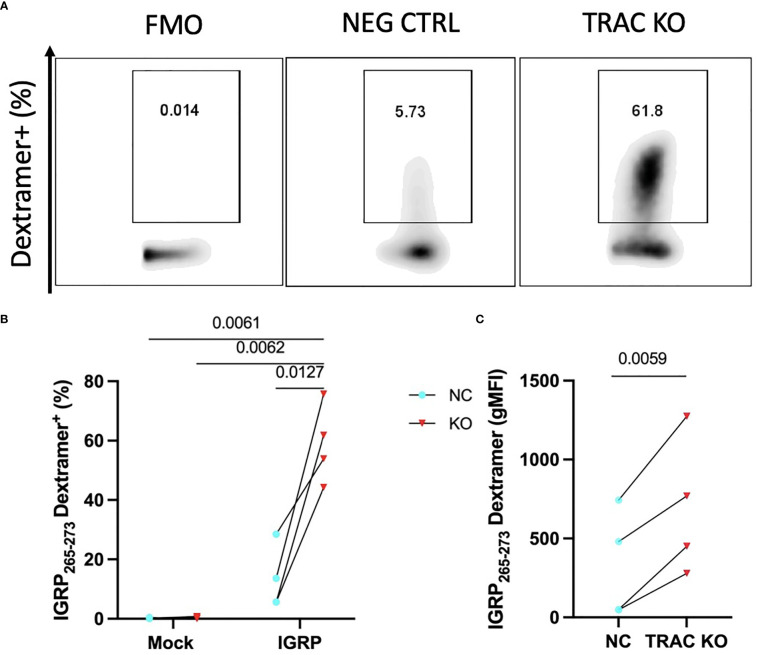
*TRAC* KO confers increased pMHC avidity. Clone 32 IGRP TCR transduced CD8^+^ T cells were stained with a pMHC-IGRP_265-273_ dextramer. **(A)** Representative plots of dextramer binding cells within the KO as compared to the negative control condition. **(B)** Significant enhancement of the dextramer binding fraction within the *TRAC* KO condition as compared to the negative control (NC; transduced with LV TCR, no endogenous *TRAC* KO) and mock conditions (electroporated with or without CRISPR but not transduced with LV TCR). Repeated measures two-way ANOVA with Tukey’s multiple comparison test, p value shown on figure (n=4). **(C)** Significant enhancement in dextramer mean fluorescence intensity (MFI) between the *TRAC* KO and NC conditions. Paired t-test, p value shown on figure (n=4).

It has been shown that competition for TCR-CD3 complex assembly between the endogenous and exogenously introduced TCR chains influences pairing and thereby, the functional avidity of each TCR dimer (28). Given that self-reactive T cells are by nature lower affinity to permit escape from thymic negative selection (29, 30), as compared to pathogen-specific T cells, mispairing could limit avidity and thus, functionality of the *de novo* autoreactive TCR. We hypothesized that deletion of the endogenous TCRα would result in an increase in avidity of the *de novo* clone 32 TCR to cognate antigen. In order to examine this, we employed peptide-major histocompatibility complex (pMHC)-dextramer reagents, which permit visualization of binding to TCR and can serve as a measure of TCR avidity, as reviewed previously (31). We expected that minimization of TCR chain mispairing would result in enhanced IGRP pMHC dextramer binding capability, as reduced dextramer binding has been shown upon TCR transduction in cells with an intact endogenous TCR (32). Indeed, staining with HLA-A2-IGRP_265-273_ dextramer revealed an increased percentage of IGRP dextramer^+^ cells in *TRAC* KO clone 32 T cell avatars (58.9 ± 13.3%) relative to clone 32 avatars that received electroporation but not sgRNA or Cas9 (negative control, 13.4 ± 10.7%, fold difference = 4.4) and mock transduced T cells lacking the *de novo* TCR and exposed to electroporation without CRISPR editing (0.21 ± 0.18%, fold difference= 276.5) and with endogenous TCR KO (0.39 ± 0.35%, fold difference = 150.1) (**Figures 2A, B**). *TRAC* KO clone 32 T cell avatars also showed increased IGRP dextramer mean fluorescence intensity (MFI) (693.3 ± 437.1) relative to negative control avatars (330.2 ± 342.2, fold difference = 2.1), both indicative of enhanced on target pMHC-TCR binding capacity (**Figure 2C**).

### 3.2 *TRAC* KO T cell avatars display increased activation in response to autoantigen

**Figure 3:**
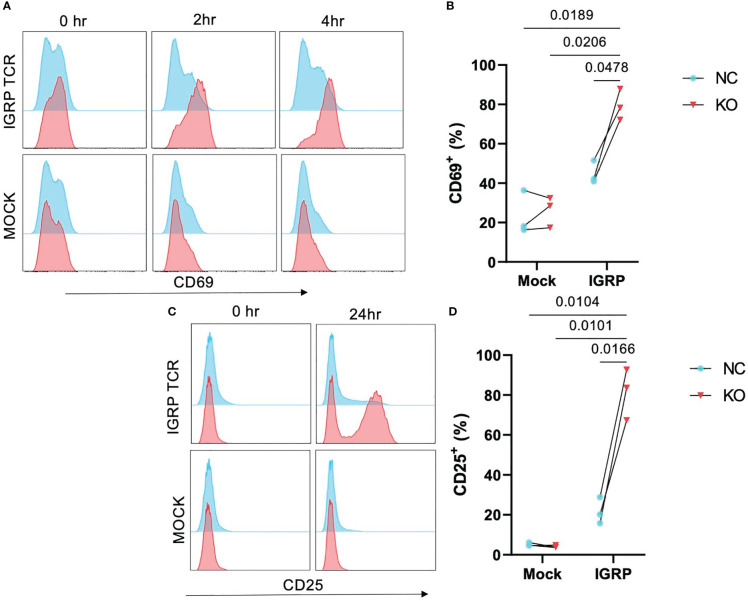
Enhanced activation in *TRAC* KO avatars. Clone 32 IGRP TCR or mock transduced CD8^+^ T cells were co-cultured at a 1:1 ratio with IGRP_265-273_ loaded HLA-A2^+^ K562 aAPCs for 0, 2, 4, and 24 hours, then assessed for activation markers CD25 and CD69 by flow cytometry. **(A)** Representative histograms of CD69 expression on *TRAC* KO IGRP TCR (upper pink) versus *TRAC* KO mock transduced (lower pink) and negative control (NC; blue) avatars at 0-, 2- and 4-hour time points. **(B)** Significant enhancement of the CD69^+^ fraction within the *TRAC* KO condition as compared to the negative control condition at 4 hours post stimulation. **(C)** Representative plots of CD25 expression on *TRAC* KO (upper pink) versus mock transduced (lower pink) and negative control (blue) avatars at 0- and 24-hour timepoints. **(D)** Significant enhancement of the CD25^+^ fraction within the *TRAC* KO condition as compared to the negative control and both mock control conditions. Repeated measures two-way ANOVA with Tukey’s multiple comparison test, p values shown on figure (n=3).

TCR affinity and functional avidity have been shown to be linearly correlated with activation by cognate antigen and downstream effector function in the context of TCR:pMHC interactions (33). We investigated whether the increased pMHC avidity observed in our *TRAC* KO avatars would translate to increased activation in response to cognate antigen. To test this, we loaded HLA-A2^+^ K562 cells with IGRP_265-273_ peptide and co-cultured these aAPCs with our *TRAC* KO or negative control avatars. We observed no significant differences in CD69 expression at 2 hours post-stimulation (**Figure S2A**), but after 4 hours of co-culture, *TRAC* KO avatars exhibited a greater percentage of cells expressing the early activation marker CD69 (79.5 ± 7.9%) relative to negative control clone 32 avatars (with intact endogenous *TRAC*, 44.8 ± 5.8%, fold difference = 1.8) and mock transduced control T cells with (26.1 ± 7.7%, fold difference = 3.0) or without *TRAC* KO (23.6 ± 11.1%, fold difference = 3.4) (**Figures 3A, B**), indicative of sustained activation in the IGRP *TRAC* KO avatars. Moreover, at the 24-hour time point, we observed an increased percentage of IGRP-reactive *TRAC* KO avatars expressing the late activation marker CD25 (81.2 ± 12.8%) as compared to IGRP-reactive negative control avatars (21.6 ± 6.7%, fold difference = 3.76) and mock transduced cells (mock *TRAC* KO: 4.1 ± 0.63%, fold difference = 19.8; mock negative control: 5.2 ± 0.83%, fold difference = 15.6) (**Figures 3C, D**). These data indicate that deletion of the endogenous TCRα permits increased activation by the *de novo* TCR.

### 3.3 *TRAC* KO T cell avatars demonstrate enhanced GZMB-dependent β-cell cytotoxicity

**Figure 4:**
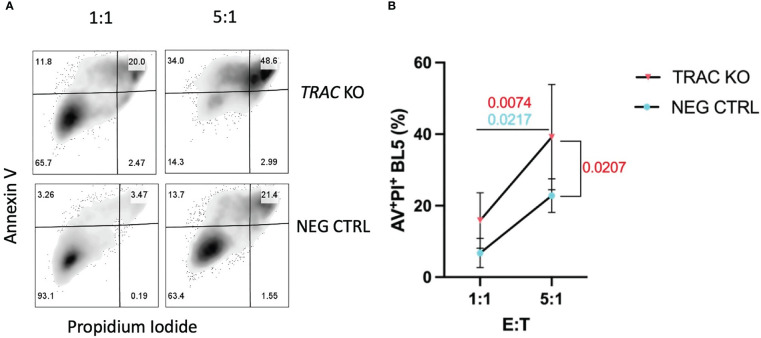
*TRAC* KO avatars display increased cytotoxicity against a human β-cell line. Clone 32 IGRP TCR transduced CD8^+^ T cells were co-cultured at a 1:1 and 5:1 effector:target (E:T) ratio with the HLA-A2^+^ βLox5 cell line for 16 hours and assessed for apoptosis and cell death with Annexin V and propidium iodide (PI) staining. **(A)** Representative contour plot of Annexin V and PI staining of βLox5 cultured with *TRAC* KO (upper) or negative control T cell avatars (lower). **(B)** Increased cell mediated lysis of βLox5 in the 5:1 vs the 1:1 effector: target (E:T) ratio for both *TRAC* KO (red, p=0.0074) and negative control (blue, p=0.0217) avatars, as well as a significant increase in killing capacity in the 5:1 E:T *TRAC* KO condition as compared to the 5:1 E:T negative control condition (red, p=0.0207). Repeated measures two-way ANOVA with Bonferroni correction, p values shown on figure (n=4).

TCR signal strength has been shown to largely influence the efficiency of cytotoxic T lymphocyte (CTL) killing (34). After demonstrating that endogenous *TRAC* KO enhanced the avidity of the clone 32 TCR, evidenced by increased pMHC-dextramer binding and activation, we reasoned that this would translate to augmented killing of IGRP-expressing target cells. We utilized the HLA-A2^+^ β-cell line βLox5 (11) as such targets in order to test the *in vitro* killing capacity of our *TRAC* KO avatars. After 16 hours of co-culture, we observed significant increases in killing between the 5:1 vs the 1:1 E:T ratios in both *TRAC* KO (*red*, p=0.0074) and negative control (*blue*, p=0.0217) conditions, as expected (**Figures 4A, B**). Importantly, we also observed a significant increase in killing capacity in the 5:1 E:T *TRAC* KO condition as compared to the 5:1 E:T negative control condition (p=0.0207) (**Figure 4B**).

**Figure 5:**
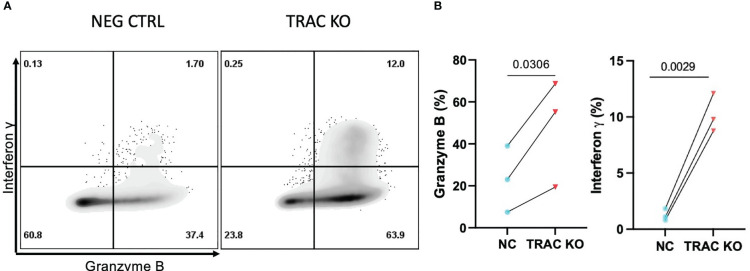
Enhanced inflammatory cytokine and cytolytic molecule production by *TRAC* KO avatars. Clone 32 IGRP TCR transduced CD8^+^ T cells were co-cultured at a 1:1 ratio with the HLA-A2^+^ K562 cell line for four hours in the presence of GolgiStop and assessed for production of effector molecules granzyme B (GZMB) and interferon-γ (IFN- γ). **(A)** Representative contour plot of IFN-γ and GZMB expression by *TRAC* KO or negative control T cell avatars. **(B)** Significant increase in the percent of cells that express GZMB (left) and IFN- γ (right) in the *TRAC* KO condition as compared to the negative control condition. Paired t test, p value shown on figures (n=3).

The mechanisms underlying CD8^+^ T cell dependent β-cell cytotoxicity have been shown to include cytolysis by perforin and granzyme B (10), as well as the induction of stress through the production of soluble effector molecules such as interferon gamma (IFN-γ) and tumor necrosis factor alpha (TNF-α) (29, 35). Thus, we assayed for the production of cytolytic molecules, namely TNF-α, IFN-γ, perforin, and GZMB, as well as IL-2 as a readout of mitogenic capacity (36), after stimulation with aAPCs presenting cognate antigen for four hours in the presence of a protein transport inhibitor. While we observed no differences in the production of TNF-α, IL-2 or perforin (**Figures S2B–D**), we observed notably enhanced production of GZMB (47.7 ± 25.5) and IFN-γ (10.2 ± 1.7) by the *TRAC* KO versus negative control (23.2 ± 15.8, fold difference = 2.1 and 1.2 ± 0.5, fold difference = 8.2, respectively) clone 32 avatars (**Figures 5A, B**). This enhancement was found to be specific to cells possessing the clone 32 TCR as there was no difference in production of these molecules between the *TRAC* KO and negative control conditions in internal control GFP- T cells (i.e., derived from the same cultures as the GFP^+^ cells but lacking the *de novo* TCR; **Figures S2E, F**).

## 4 Discussion

In our study, we utilized CRISPR/Cas9 to delete the endogenous TCRα of engineered autoreactive CD8^+^ T cell avatars, as has been previously performed with LV transduced cancer-reactive T cells (22). To our knowledge, ours is the first study to illustrate these improvements in T cell avidity and cytotoxic function as it pertains to β-cell antigen specificity, though others have utilized a similar approach when designing chimeric antigen receptor (CAR) regulatory T cells (Tregs) targeting the HLA-A2 molecule (37). We first demonstrated the utility of this approach in enhancing the ability of the *de novo* IGRP-reactive TCR to bind pMHC-dextramer reagents. While dextramer reagents demonstrate increased capacity for binding lower affinity TCRs than tetramer reagents (38), previous studies have shown that the pMHC-TCR affinity threshold for multimer binding to be lower than that required for activation (39), resulting in the possibility for low affinity and recently activated T cells which have downregulated the CD3/TCRαβ complex (40) to be underestimated using this technique. However, enhancing the capacity of the *de novo* TCR to bind these reagents through endogenous *TRAC* KO could be helpful in a clinical setting to monitor the persistence of an ACT product. We also demonstrated that deletion of the endogenous TCRα served to augment the activation of our T cell avatars and consequently, the killing capacity of a human β-cell line. We note that at the earlier activation timepoint (2 hours) there was no difference in activation, while at the later timepoints (4 and 24 hours) we observed increased expression of activation markers CD69 and CD25, respectively. Future experiments will be required to determine the impact on TCR activation kinetics and to elucidate if the resulting upregulation of activation markers is the consequence of increased magnitude or duration of activation, or both. Furthermore, while the TCR utilized in this manuscript has been shown to be reactive to IGRP_265-273_, it has also been indicated that this receptor might possess promiscuity towards other epitopes presented in the context of HLA-A2 (7). While a certain degree of cross-reactivity likely is advantageous in that the limited TCR repertoire within an individual can provide protection against a vast array of foreign exposures, it is possible that this promiscuity may also contribute to autoreactivity. Indeed, studies examining autoreactive receptors have shown promiscuity to a wide variety of endogenous and foreign epitopes (41, 42). One particular clone of note is the 1E6 TCR clone, which is reactive to PPI_15-24_ and has been shown to mediate β-cell killing (35). In a comprehensive set of experiments testing TCR degeneracy, this clone was shown to recognize more than one million peptides in the context of HLA-A2 (41). Thus, it is apparent that cross-reactivity could be a key feature of autoreactive receptors and an important mechanism of autoimmunity that warrants further investigation. With isogenic systems becoming an attractive means to model diseases (11), we propose that the use of gene edited T cell avatars, such as those presented herein, will aid in deciphering the mechanisms underlying dysregulation in the form of cell-cell interactions, chemotaxis, and phenotype and function of antigen specific T cells. As such, experiments are ongoing to track the movement of these T cells within an engineered islet niche (43). Further genetic engineering of T cell avatars and their targets will provide options to modulate key candidate genes and single nucleotide polymorphisms (SNPs) (10, 39) predicted to be important for phenotypes, immune cell or otherwise.

Though islet antigen specific CD8^+^ T cells have been implicated as key players in T1D disease pathogenesis (6, 44), we still know little about their phenotype and function *in vivo*. Data resulting from clinical trials support the critical need to understand CD8^+^ T cell signatures in order to unravel mechanisms central to autoimmune β-cell destruction. In particular, response signatures of CD8^+^ T cell exhaustion following teplizumab therapy (45) would indicate that inducing this phenotype could preserve β-cell function. However, other studies have observed human T1D antigen specific CD8^+^ T cells as having a “self-renewing” stem cell memory phenotype (46, 47), which would indicate that these cells are not as susceptible to exhaustion or tolerization as one would expect from cells that are chronically exposed to antigen (48). Bridging these gaps in understanding necessitates optimal *in vitro* modeling of autoreactive CD8^+^ T cell function to identify targetable molecules and pathways to reduce immune-mediated pathology through pharmacological or biological interventions.

ACT are at the forefront of clinical innovation, with an increased focus on genetic engineering to enhance the efficacy and safety of these cell products. In T1D, there is great interest in developing antigen-specific Treg ACT products (49, 50). Islet antigen-specific Tregs have the potential to suppress diabetogenic T cells *in vitro (* 24) and in mouse models of T1D (51), but these cells have yet to effectively be used to combat disease in humans. Optimizing the activation of Tregs specific for β-cell antigens is of particular importance, as we have previously shown that engineered GAD_555-567_ specific Tregs possessing the higher affinity clone R164 TCR outperformed the lower affinity clone 4.13 TCR Tregs, which recognize the same peptide, in antigen-specific suppressive capacity (24). While both low- and high-avidity TCR interactions in Tregs appear to be important for suppression of autoimmunity (52), Tregs possessing higher affinity receptors have been shown to preferentially home to the pancreatic lymph node and islets, and express inhibitory receptors to a greater degree than lower affinity Tregs (52). Therefore, enhancing the avidity of an antigen-specific Treg cell product via endogenous *TRAC* KO may confer several functional advantages.

### 4.1 Limitations of the study

Our study focuses on a single HLA class I restricted receptor, thus the impact of endogenous TCR deletion on receptors of different specificities, HLA restrictions, and affinities in T1D is unknown, and though others have seen success with higher affinity tumor-reactive receptors (21, 22), recapitulating the phenotype and function of a lower affinity receptor may be more challenging. Additionally, we utilized an immortalized β-cell line, which is not functionally identical to primary human islets, thus future efforts testing this workflow with primary human islet material will be necessary, particularly for specificities such as derivatives of preproinsulin, or post-translationally modified epitopes (14). Moreover, in our study we have targeted the *TRAC* locus, as the paired TCRαβ dimer is necessary for full function of the TCR (53), though we acknowledge the possibility of residual mispairing between the endogenous β- and *de novo* α-chain, as well as potential alloreactivity due to incomplete HLA matching. Additionally, the field of genome editing is rapidly evolving, and newer methodologies allowing for targeted TCR delivery and regulation via the endogenous promoter (54), as well as base editing strategies enabling induction of a stop codon with minimal DNA damage (55), though lower efficiency at the time of writing, may soon provide more context appropriate results or products for ACT. We also recognize that utilizing humanized animal models or cell and tissue engineering strategies will likely be necessary to confirm *in vivo* functionality (56) and obtain a complete picture of islet-immune interactions (43). These studies may include investigating autoantigen specific T cell function, homing, and long-term engraftment. Along a similar vein, T1D is a highly polygenic disease (57), and this study was not designed to investigate the influence of T1D risk loci on T cell avatar function, which is known to alter TCR signals (25). We are currently working to establish isogenic systems in order to appropriately test the contributions of key SNPs which may modulate TCR signaling and cytotoxic T cell function (11).

## Data availability statement

The raw data supporting the conclusions of this article will be made available by the authors, without undue reservation.

## Ethics statement

Ethical review and approval was not required for studies using purchased human blood products in accordance with the local legislation and institutional requirements. Written informed consent for participation was not required as these studies were conducted using blood products acquired from LifeSouth Blood Centers under Institutional Review Board exempt protocols in accordance with the national legislation and institutional requirements.

## Author contributions

LDP designed the studies, conducted experiments, acquired data, analyzed data, acquired funding, and wrote the manuscript. W-IY designed the studies, conducted experiments, acquired data, analyzed data, and reviewed/edited the manuscript. JMA conducted experiments, acquired data, and reviewed/edited the manuscript. MEB conducted experiments, acquired data, and reviewed/edited the manuscript. ALP contributed to discussion and reviewed/edited the manuscript. CEM contributed to discussion and reviewed/edited the manuscript. TMB conceptualized the project, designed studies, provided reagents, acquired funding, and reviewed/edited the manuscript. All authors contributed to the article and approved the submitted version.
