# Supplementary material for: Modeling cell-mediated immunity in human type 1 diabetes by engineering autoreactive CD8+ T cells
Source: Front Immunol. 2023 May 30;14:1142648. doi: 10.3389/fimmu.2023.1142648 (PMC10262917; doi:10.3389/fimmu.2023.1142648)
Supplement: Supplementary file 1 [file DataSheet_1.docx]

Supplementary Material


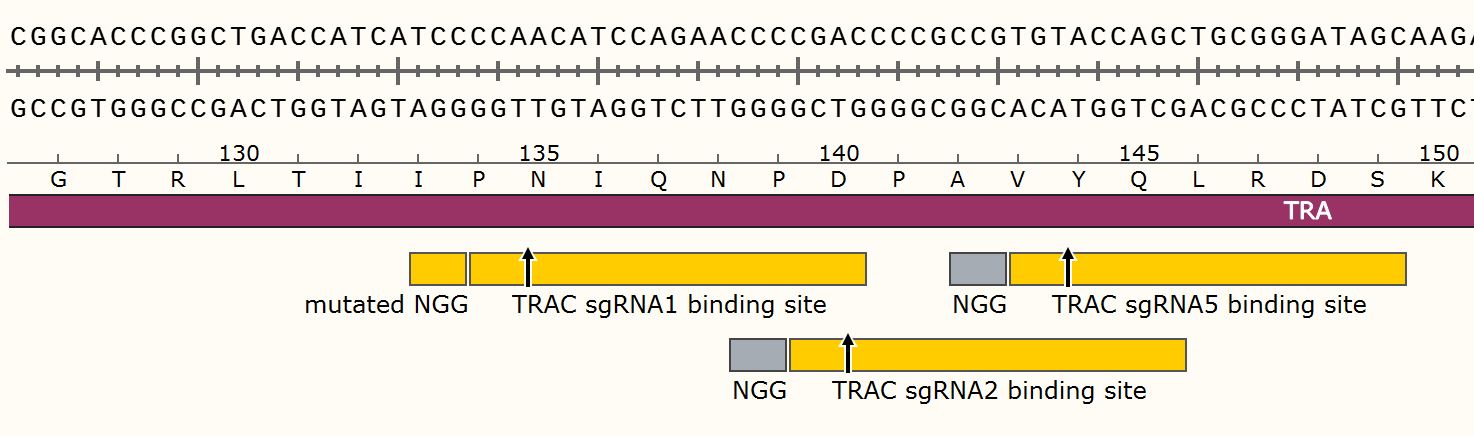


**Supplementary Figure 1: sgRNA sequences information relative to the clone 32 construct.** Shown are the top 3 performing guide sequences identified through the Synthego bioinformatic design tool. We opted for sgRNA1 for our studies (sequence:GUCAGGGUUCUGGAUAUCUG, genomic cut site *TRAC* Exon 1 22,547,506) as our construct possessed a mutated PAM as well as 4 bp mismatches in the sgRNA binding site.


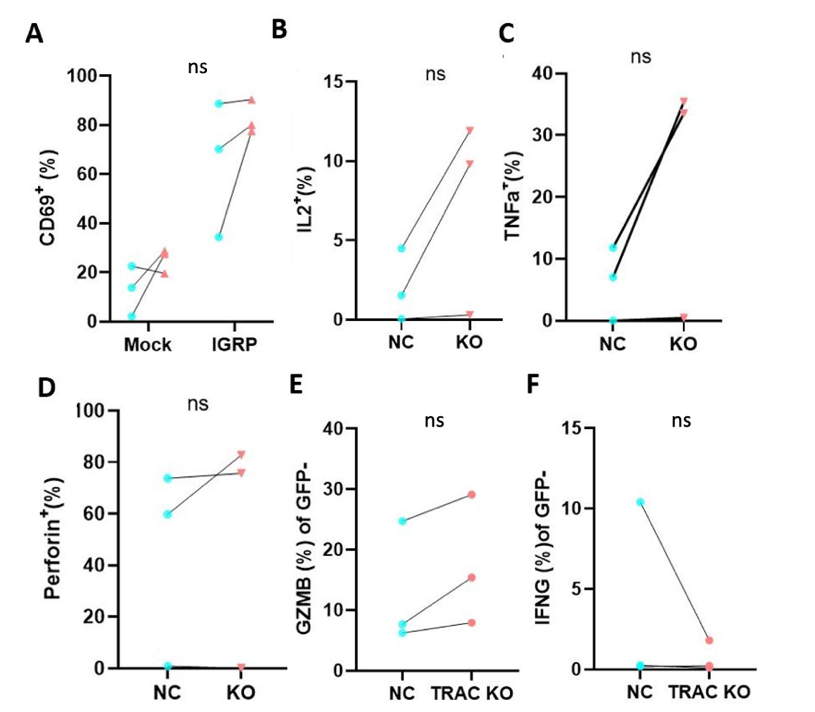


**Supplementary Figure 2: Non-significant differences between *TRAC* KO and NC avatars post-activation.** IGRP clone 32 T cell avatars were co-cultured at a 1:1 ratio with the HLA-A2^+^ K562 cell line and assessed for differences in extracellular activation marker expression or for production of cytokines and effector molecules as described in methods. A) We observed no differences in CD69 expression between edited and unedited T cell avatars at 2 hours post-activation. We also observed no differences in production of IL-2 (B), TNF-a (C) or Perforin (D), and confirmed that edited cells which did not possess the de novo TCR (GFP-) did not produce more GZMB (E) or IFNG (F) after activation.
